# LL37 and Cationic Peptides Enhance TLR3 Signaling by Viral Double-stranded RNAs

**DOI:** 10.1371/journal.pone.0026632

**Published:** 2011-10-21

**Authors:** Yvonne Lai, Sreedevi Adhikarakunnathu, Kanchan Bhardwaj, C. T. Ranjith-Kumar, Yahong Wen, Jarrat L. Jordan, Linda H. Wu, Bogdan Dragnea, Lani San Mateo, C. Cheng Kao

**Affiliations:** 1 Department of Molecular and Cellular Biochemistry, Indiana University, Bloomington, Indiana, United States of America; 2 Discovery Research, Centocor Research and Development, Inc., Radnor, Pennsylvania, United States of America; 3 Department of Chemistry, Indiana University, Bloomington, Indiana, United States of America; University of Texas Medical Branch, United States of America

## Abstract

**Background:**

Toll-like Receptor 3 (TLR3) detects viral dsRNA during viral infection. However, most natural viral dsRNAs are poor activators of TLR3 in cell-based systems, leading us to hypothesize that TLR3 needs additional factors to be activated by viral dsRNAs. The anti-microbial peptide LL37 is the only known human member of the cathelicidin family of anti-microbial peptides. LL37 complexes with bacterial lipopolysaccharide (LPS) to prevent activation of TLR4, binds to ssDNA to modulate TLR9 and ssRNA to modulate TLR7 and 8. It synergizes with TLR2/1, TLR3 and TLR5 agonists to increase IL8 and IL6 production. This work seeks to determine whether LL37 enhances viral dsRNA recognition by TLR3.

**Methodology/Principal Findings:**

Using a human bronchial epithelial cell line (BEAS2B) and human embryonic kidney cells (HEK 293T) transiently transfected with TLR3, we found that LL37 enhanced poly(I:C)-induced TLR3 signaling and enabled the recognition of viral dsRNAs by TLR3. The presence of LL37 also increased the cytokine response to rhinovirus infection in BEAS2B cells and in activated human peripheral blood mononuclear cells. Confocal microscopy determined that LL37 could co-localize with TLR3. Electron microscopy showed that LL37 and poly(I:C) individually formed globular structures, but a complex of the two formed filamentous structures. To separate the effects of LL37 on TLR3 and TLR4, other peptides that bind RNA and transport the complex into cells were tested and found to activate TLR3 signaling in response to dsRNAs, but had no effect on TLR4 signaling. This is the first demonstration that LL37 and other RNA-binding peptides with cell penetrating motifs can activate TLR3 signaling and facilitate the recognition of viral ligands.

**Conclusions/Significance:**

LL37 and several cell-penetrating peptides can enhance signaling by TLR3 and enable TLR3 to respond to viral dsRNA.

## Introduction

Innate immune receptors provide our first line of defense against invading microbes and are essential for activating adaptive immune responses [1]. The eleven Toll-like receptors (TLRs) in the human genome recognize a variety of ligands that possess molecular signatures identifying them as non-self molecules [2], resulting in induction of cytokines that modulate both anti-pathogen and adaptive immune responses [1], [3].

During viral infection, tissue injury or inflammation, dsRNA released by viruses or necrotic cells could activate TLR3, leading to translocation of transcription factors NF-ΚB, and IRF3 into the nucleus, modulation of gene expression and increased secretion of type I interferons and inflammatory cytokines, as well as the maturation of dendritic cells [1]. Single nucleotide polymorphisms in TLR3 leading to inappropriate TLR3 expression or defective signaling are linked to increased severity of human herpesvirus and influenza virus infection and age-related macular degeneration [4], [5], [6]. Moreover, TLR3 knock-out mice have an impaired response to cytomegalovirus infection, suggesting that TLR3 plays an important role in the defense against viral infection [7]. Recognition of auto-antigens may lead to over-activation of TLR3, resulting in chronic inflammation [8], [9], [10]. TLR3 agonists are also efficacious vaccine adjuvants [11] and have shown promise in inducing apoptosis of cancer cells [12]. All of these properties make TLR3 an important therapeutic target for multiple diseases. However, effective therapies will require a better understanding of TLR3 functions, including its recognition of dsRNA ligands.

Poly(I:C), a synthetic dsRNA analog, is frequently used as a TLR3 ligand [13] and has been co-crystallized with the TLR3 ectodomain [14]. RNAs extracted from necrotic cells and siRNAs of nonspecific sequences have been reported to activate TLR3 [15], [16]. However, purified RNAs from necrotic cells and siRNA are unable to induce TLR3 in a number of human cell lines (Lai Y, unpublished observations). In addition, most homopolymeric dsRNA or viral RNAs fail to activate TLR3 [17]. We hypothesize that more complex RNAs will require additional factors before they can induce signaling by TLR3.

LL37 is a human cationic antimicrobial peptide that enters cells to act on multiple TLRs [18], [19], [20], [21]. It is released primary from neutrophils [22] and is cleaved from the C-terminal portion of hCAP-18 by proteinase 3. Improper levels of LL37 are associated with chronic respiratory diseases [23] and autoimmune diseases such as psoriasis [24], [25], [26]. Intriguingly, LL37 interacts with several classes of TLR ligands to modulate signaling by various TLRs. It can complex to bacterial lipopolysaccharide (LPS) to prevent activation of TLR4 [27], to single-stranded (ss) DNA to enhance signaling by TLR9 [25], [27], and to ssRNA to enhance signaling by TLR7 and 8 [26]. LL37 can also synergize with flagellin to regulate TLR5 and with PAM3CSK4 to modulate TLR2/1 [28]. At higher concentrations (5 to 10 µM), LL37 induces IL6 production in transformed human bronchial epithelial cells [29]. Most relevant to the present study, LL37 can act in concert with the TLR3 agonist poly(I:C) to increase IL8 and or IL6 production [28]. It is not clear how LL37 enhances TLR3 signaling, although Filewood et al [28] observed significant cytotoxicity that accompanied the enhancement of poly(I:C) signaling by LL37.

In this study we show that LL37 enables viral dsRNAs to serve as agonists for TLR3, in addition to poly(I:C). LL37 enhances cytokine responses in rhinovirus infected BEAS2B cells and in peripheral blood mononuclear cells (PBMCs) induced by poly(I:C) in a TLR3-dependent manner. LL37 also co-localizes with TLR3 and dsRNA in BEAS2B cells, suggesting the formation of a RNA-receptor-protein complex. *In vitro,* LL37 complexes with dsRNA and also alters the conformation of dsRNA, potentially facilitating recognition by TLR3. Furthermore, we identified several cell penetrating peptides that activate TLR3 signaling in response to dsRNAs without affecting the LPS-dependent signaling by TLR4.

## Results

### LL37 can enhance poly(I:C) signaling

**Figure 1:**
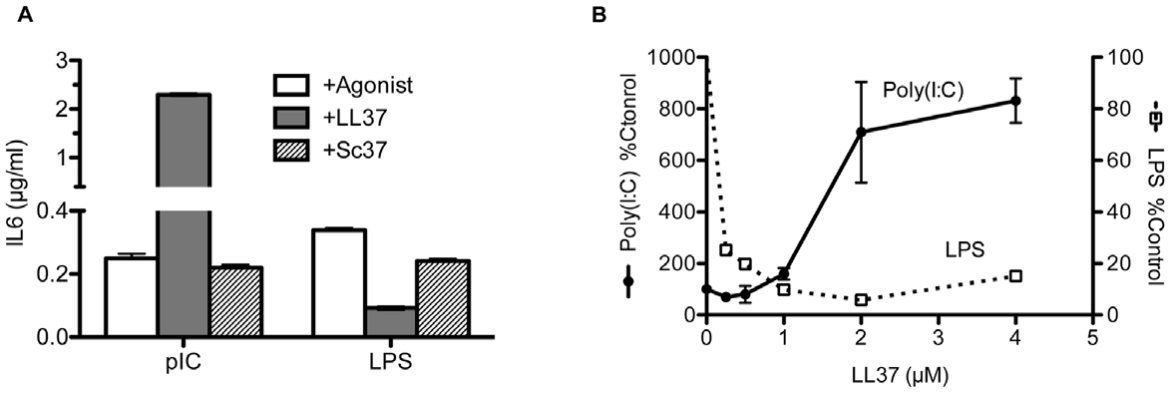
Effects of LL37 on cytokine production by BEAS2B cells. A) LL37 can enhance IL6 production in response to dsRNA and repress IL6 levels in response to LPS. Cells were stimulated with agonists ± LL37 or scrambled LL37 (Sc37). After 20 h, the culture media were collected. An aliquot of the media was assessed for secreted IL6 using a human IL6 ELISA assay and the amount of IL6 normalized to total volume. The final concentrations of LL37, Sc37, poly(I:C) and LPS were 2.2 µM, 2.2 µM, 0.13 µg/ml and 1 µg/ml, respectively. LL37 enhanced poly(I:C) induced IL6 production (p<0.0001;) while Sc37 had no effect (p = 0.2). B) Dose-dependent effects of LL37 on poly(I:C)-induced and LPS-induced IL6 levels. Culture media were harvested 20 h after the addition of ligands and proteins. Each sample was performed in duplicate or triplicate and data plotted as mean ± SEM.

Bronchial epithelial cells are the first line defense against foreign microbes in our respiratory system and initiate immune response by producing cytokines and chemokines, resulting in recruitment of inflammatory cells [30]. The human bronchial epithelial cell line, BEAS2B, which endogenously expresses TLR3, 4, 9, and RIG-I has been extensively used to study TLR3 function [31] and was used in this study to examine whether LL37 modulates TLR3 signaling. Since LL37 and TLR3 ligands may likely encounter TLR3 after endocytosis of materials outside of cells, adding them to the media of cultured cells should be a suitable model to study the roles of LL37 and dsRNAs. We note that this mode of uptake does not activate cytoplasmic innate immune receptors, such as RIG-I like receptors, which requires transection of the ligands [32]. In the absence of TLR3 agonist poly(I:C), the basal level of IL6 was at 0.06 ± 0.10 µg/ml (n = 32). Addition of poly(I:C) to the medium increased IL6 levels 15 ± 2.2 fold (n = 12) above basal levels (Fig. 1A) [31]. LL37 (2.2 µM) further enhanced poly(I:C)-induced IL6 levels by an average of 4.5 ± 0.67 fold (n = 23; p<0.0001) when compared to poly(I:C) alone. A peptide with the scrambled LL37 sequence (Sc37) did not enhance IL6 production (Fig. 1A; n = 7; p = 0.2). Similar results were observed for LL37 and Sc37 with IL8 (data not shown). LL37 also enhanced the poly(I:C)-induced increase in IL6 and interferon beta (IFNβ) mRNAs as determined by RT-PCR (Figure S1; p<0.05 for both mRNAs).

Consistent with previous reports, we found that LL37 inhibited LPS-induced IL6 production (Figures 1A and 1B) [21]. The EC_50_ for LL37 enhancement of poly(I:C)-induced IL6 production was ∼1.5 µM and the IC_50_ for inhibiting LPS-dependent IL6 production was less than 0.5 µM (Figure 1B). LL37 had a minimal effect on IL6 production from 0-10 µM (when it was added to cells in the absence of poly(I:C) (1.4 ± 0.2 fold above basal IL6 levels at 3 µM; n = 13; p>0.5). It also had minimal effects on the BEAS2B cell viability at concentrations at or less than 3 µM, either by itself or in the presence of poly(I:C) (Figure S2; p>0.5 for 2.5 µM LL37 ± poly(I:C), n = 5).

### LL37 acts on TLR3 signaling

**Figure 2:**
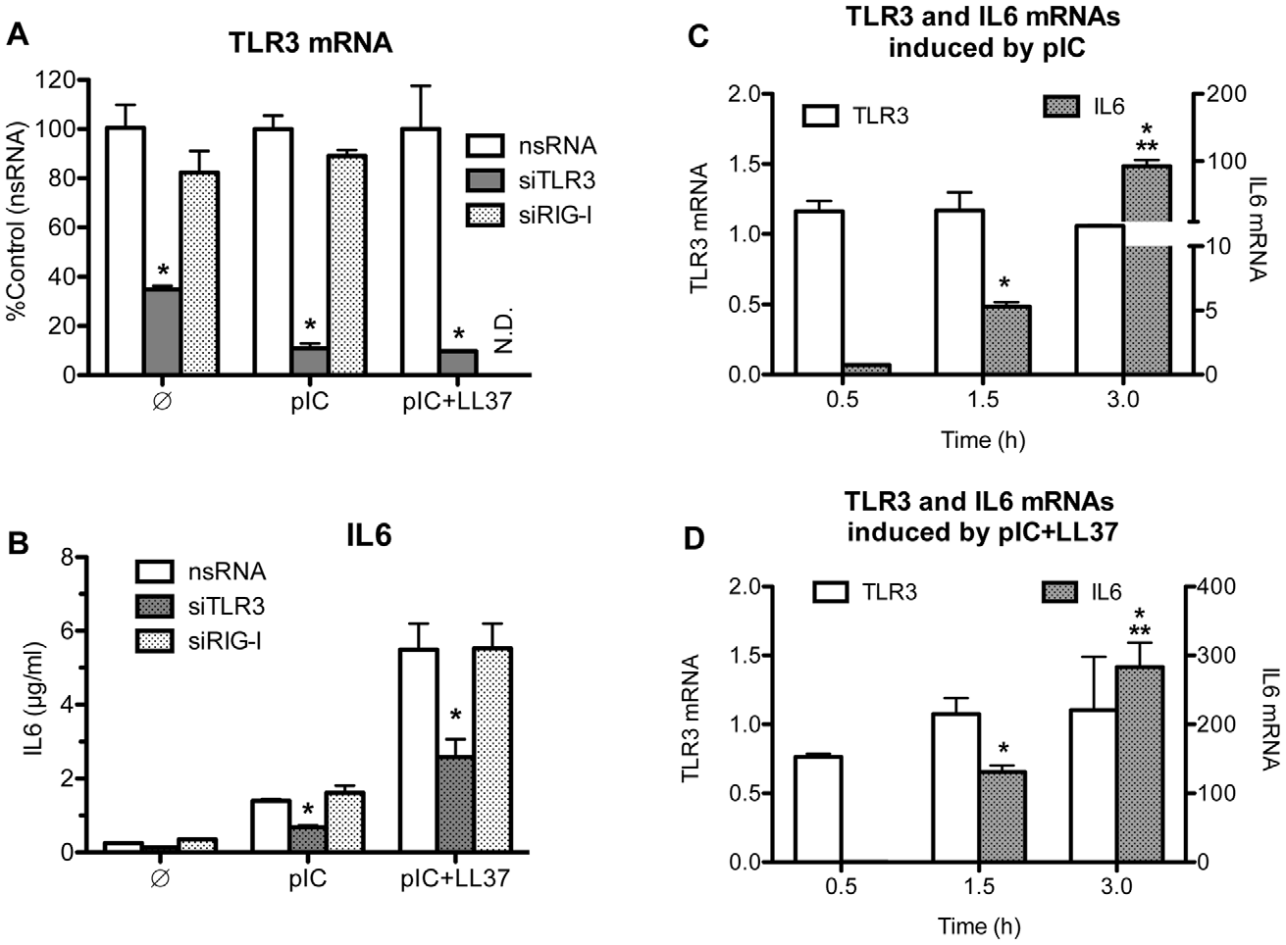
TLR3 is responsible for the enhancement of IL6 production observed in BEAS2B cells. A) A demonstration that a set of three siRNAs to TLR3 (siTLR3) can reduce TLR3 mRNA in BEAS2B cells. siRNAs to RIG-I (siRIG-I) and a nonspecific siRNA (nsRNA) were used as controls. All siRNAs were used at 30 nM. 48 h after transfection with siRNAs, cells were either not stimulated (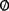) or stimulated with either poly(I:C) (0.13 µg/ml; pIC) or poly(I:C)+LL37 (3 µM) (pIC+LL37). After 20 h, total RNA was extracted and RT-PCR was performed using primers specific for TLR3 and GAPDH. Data is presented as %Control using corresponding agonist treatment. * Indicates p<0.05 compared to control. B) siRNAs to TLR3 reduced IL6 production induced by poly(I:C) or poly(I:C)+ LL37. The cells were transfected with siRNAs to TLR3 (siTLR3), siRNA to RIG-I (siRIG-I) or a control siRNA as described in A. The culture media were harvested 20 h after the addition of the ligands and the level of secreted IL6 protein determined. Each sample was performed in triplicates and the mean ± SEM shown. * Indicates p<0.05 compared nsRNA in corresponding treatment with agonist. C& D) The abundances of IL6 (right y-axis) and TLR3 messages (left y-axis) in response to the addition of poly(I:C) (C) or poly(I:C)+LL37 (D). The samples used were harvested after poly(I:C) addition to the BEAS2B cell culture media at the times specified in the horizontal axis. The RNAs were then subjected to RT-PCR as described in the materials and methods using either primers specific for IL6 or for TLR3. * Indicates p<0.05 compared to no treatment and ** indicates p<0.05 compared to poly(I:C) treatment. There is no difference (p>0.5) for TLR3 mRNA among any of the treatments or between any of the time points (0.5–3 h).

Both TLR3 and RIG-I are activated by poly(I:C) [33]. Transfection of RNA agonists into the cell's cytoplasm activates cytoplasmic RIG-I while addition of dsRNA agonists to cell media and subsequent uptake of agonists by endocytosis activates TLR3 [32]. To confirm that TLR3 was required for LL37-mediated enhancement of IL6 production, we knocked down TLR3 or RIG-I expression with siRNAs (Figure 2A). RT-PCR was used to confirm that the siRNAs targeting TLR3 selectively reduced TLR3 message (Figure 2A). TLR3 mediates the LL37 effect as treatment of BEAS2B cells with TLR3 siRNAs reduced IL6 levels by 51 ± 3% and 51 ± 9%, in cells that had been treated with poly(I:C) alone or poly(I:C) plus LL37 as compared to a control siRNA (Figure 2B; reduction p<0.02 for basal level, treatment with poly(I:C) or poly(I:C)+LL37). In contrast, siRNA to RIG-I minimally affected IL6 levels (16 ± 13% and 0 ± 12%, for treatment with poly(I:C) alone or with LL37 respectively; p>0.2) (Figure 2B; n = 3). In control experiments we demonstrated that siRNA to RIG-I decreased RIG-I message by more than 80% (Figure S3), but did not affect IL6 levels when the poly(I:C) was added to the medium of the BEAS2B cells (Figures 2B and S3, p>0.5). Together these results demonstrate that TLR3, but not RIG-I, is required for the LL37-dependent enhancement of IL6 production in BEAS2B cells.

The presence of LL37 and poly(I:C) could affect either TLR3 signaling or its expression. To address these two possibilities, we used RT-PCR to examine the mRNA levels of IL6, and TLR3 within three hours after the introduction of agonists. Poly(I:C) or poly(I:C) and LL37 (3 µM) did not detectably change the level of the TLR3 mRNA within the first three hours (p>0.5), during which time IL6 mRNA levels were significantly increased (Figures 2C&D, p<0.05). The levels of IL6 continued to increase and began to plateau after 10 h after ligand addition (data not shown). Together these results suggest that the change we observed with IL6 is due to activation of the TLR3 signaling pathway rather than transcriptional activation of TLR3.

### Characteristics of RNAs that can interact with LL37 to modulate TLR3 signaling

**Figure 3:**
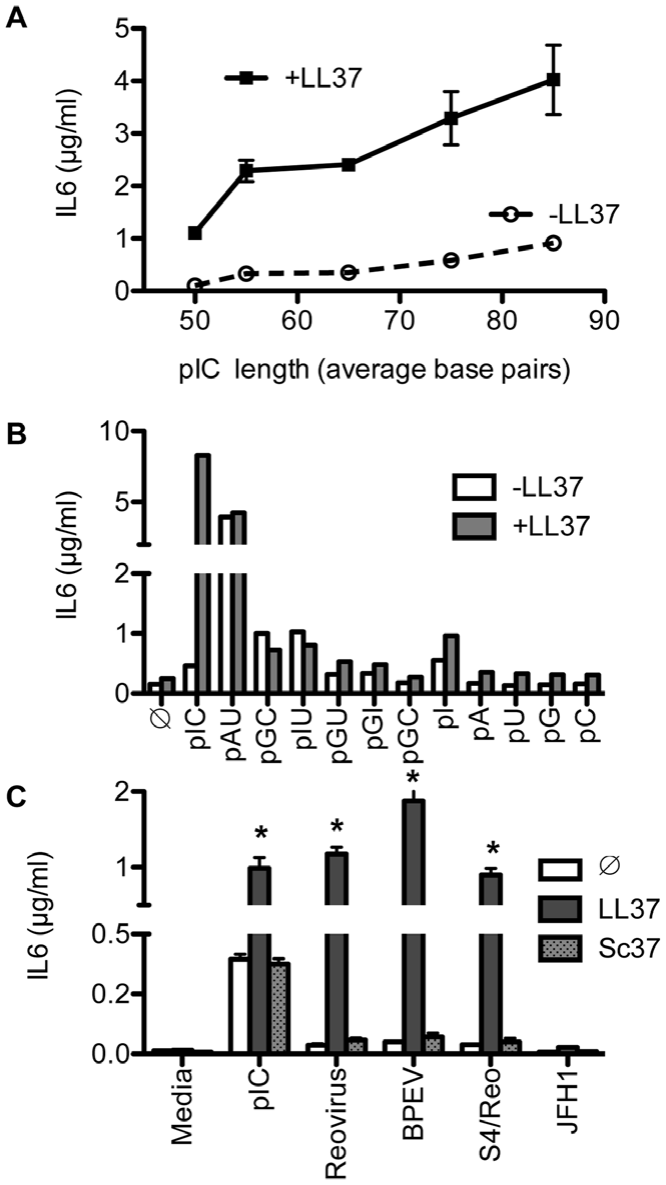
Effects of homopolymeric RNAs and viral dsRNA on IL6 production by BEAS2B cells. A) LL37 enhancement of IL6 production increased with increasing length of poly(I:C). Size-selected poly(I:C) was added to BEAS2B cells at 0.5 µg/ml ± 2.2 µM LL37. B) Effect of homopolymeric single or double-stranded RNAs on TLR3 signaling. BEAS2B cells were either untreated (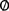) or induced by the single or double-stranded RNAs (0.5 µg/ml) ± LL37 (2.2 µM). Culture media were harvested 24 h after ligand addition and IL6 levels in the medium were quantified by ELISA. C) LL37 significantly enhanced IL6 production induced by viral dsRNAs. The dsRNAs were the genomic RNAs from Reovirus, extracts of plants expressing high levels of the Bell pepper endornavirus (BPEV), and Reovirus-derived S4 dsRNA (S4/Reo). The ssRNA from Hepatitis C virus JFH1 served as a control. Where present, LL37 and Sc37 were both at a final concentration of 2.2 µM. *Indicates p<0.05 compared to RNA ligand alone.

We next sought to better define the RNA features that determine its interactions with LL37 to modulate TLR3 signaling. A feature of poly(I:C)-dependent TLR3 activation is that the minimal length for stable binding is ∼50 bp [34]. In HEK 293T cells transiently transfected with TLR3 (293T/TLR3), TLR3 signaling responds to ligands of ∼85 bp [35]. To test whether LL37 affects the length of poly(I:C) recognized by TLR3, size-fractionated poly(I:C) preparations were used as TLR3 ligands in BEAS2B cells (Figure 3A). In the presence of LL37, poly(I:C) of 50 to 85-bp in length significantly increased IL6 production in a length-dependent manner (P<0.004, n = 3; Figure 3A).

Several single and homopolymeric dsRNAs were examined to determine the influence of base composition on the enhancement of TLR3 signaling by LL37. Poly(G:C), poly(I:U), poly(G:U) or poly(G:I) or single-stranded poly(I), poly(A), poly(U), poly(G), poly(C) all had modest and variable effects on IL6 production in the absence of LL37. In the presence of LL37, slight increases in IL6 levels were observed (Figure 3B). Poly(A:U) did robustly increase IL6 levels in BEAS2B cells in the absence of LL37 and this increase was sensitive to siRNA knockdown of TLR3 (data not shown). However, LL37 did not further enhance poly(A:U)-induced signaling (Figure 3B). These results show that the base composition and the length of the RNAs influence the LL37-dependent enhancement of TLR3 signaling. In addition, LL37 significantly enhanced TLR3 signaling only with poly(I:C), and had only modest or no observable effects with the other homopolymeric dsRNAs.

### LL37 enhanced TLR3 signaling by viral dsRNAs

To examine whether LL37 could affect TLR3 signaling in response to viral RNAs, we tested dsRNAs extracted from Reovirus and Bell pepper endornavirus (BPEV). We also included ssRNA from Hepatitis C virus strain JFH1 (Figure 3C) as an example of viral ssRNA even though BEAS2B cells could not replicate HCV RNA. In the absence of LL37, poly(I:C) was the only dsRNA that resulted in robust IL6 production (Fig. 3C). Reovirus dsRNA, BPEV dsRNA, and JFH1 ssRNA only induced IL6 levels by 2 ± 0.7 (n = 8), 1.7 ± 0.5 (n = 5), and 1.4 ± 0.5 (n = 4) fold, respectively, above basal levels (Figure 3C) though inductions were not statistically significant (p>0.5) for all three RNAs. However, the addition of LL37 (2 µM) dramatically increased IL6 production by the dsRNAs from Reovirus (12.4 ± 5 fold; n = 5; p = 0.004) and BPEV (25.5 ± 9 fold; n = 5; p<0.05) to levels comparable to that of cells treated with poly(I:C) and LL37. In contrast, the ssRNA from JFH1 virus did not significantly affect IL6 production (Figure 3C; n = 4, p>0.5). Sc37 did not increase IL6 production by any of the viral RNAs tested (Figure 3; p>0.5, n>4). These results show that LL37 can mediate recognition of two different viral dsRNAs.

The viral dsRNAs were purified from virions or infected tissues while the JFH-1 RNA was transcribed *in vitro*. This difference prompted us to examine whether *in vitro* transcribed dsRNA can be recognized by TLR3 in the presence of LL37. Annealed transcripts of the sense and antisense strands of the S4 Reovirus RNA of about 1100-bp minimally enhanced IL6 secretion in the absence of LL37 (0.2 ± 0.04 µg/ml; p = 0.02, n = 14). However, the addition of LL37 greatly enhanced S4-induced IL6 production (22 ± 5 fold, p<0.0001, n = 18) (Figure 3C). siRNAs to TLR3 attenuated the enhancement of dsRNA-induced signaling by LL37 (53% inhibition for Reovirus dsRNA+LL37, n = 2; data not shown), confirming that IL6 production was mediated by TLR3. Furthermore, the extent of S4-dependent signaling was similar to that for dsRNA purified from Reovirus virions, suggesting that postranscriptional modifications of the viral RNAs are not required for LL37 to enhance TLR3 signaling.

### LL37 enables TLR3 to respond to viral dsRNAs in HEK 293T/TLR3 cells

**Figure 4:**
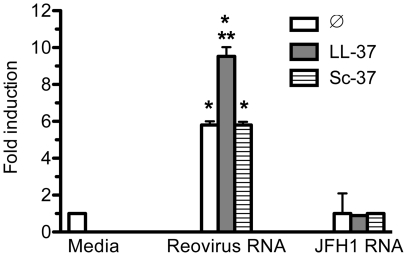
LL37 enhanced dsRNA-induced signaling in 293T cells transiently expressing TLR3. 16 h after transfection with plasmids to express TLR3 and the luciferase reporters, reovirus dsRNA or JFH1 ssRNA (both at 1 µg/ml) was added to the media of the transfected cells ± 2 µM of LL37 or Sc37. The cells were analyzed for luciferase activity 20 h after induction using the normalized ratio of firefly/*Renilla* luciferase activities. The data are presented as fold induction over media control. Each bar shows mean (±SEM) of three independent experiments and the results are representative of more than five independent experiments. *Indicates p<0.05 compared to basal level of reporter activity while ** indicates p<0.05 compared to reporter activity in the presence of reovirus dsRNA.

HEK293T (293T) cells transiently transfected to express TLRs and an interferon stimulated response element (ISRE) reporter-driven firefly luciferase have been extensively used to study TLR signaling [36]. 293T cells transiently transfected with TLR3 (293T/TLR3) did have a modest response in reporter activity in the presence of Reovirus dsRNA (5.8 ± 0.2 fold above the mock-treated control; p<0.005, n = 8) (Figure 4). LL37 (2 µM) increased reporter levels by an additional 1.7-fold above that of the Reovirus dsRNAs (Figure 4; p<0.0005, n = 8). The control reaction with JFH1 ssRNA had no effect on reporter levels whether or not LL37 was present (p>0.5). In addition, Sc37 also did not affect reporter activity from 293T/TLR3 cells treated with Reovirus dsRNA (Figure 4, p = 0.5, n = 4). Reporter activity in 293T cells transfected with vector or RIG-I was not increased by the addition of Reovirus dsRNA or JFH1 ssRNA in the culture media (data not shown). Taken together these results show that LL37 can enhance TLR3 signaling by viral dsRNAs in 293T/TLR3 cells. We note, however, that while 293T/TLR3 cells can respond to poly(I:C), exogenously-provided LL37 did not further increase poly(I:C)-induced reporter activity despite numerous attempts (data not shown). These results show that there are subtle differences in the effects of ligands in 293T/TLR3 cells when compared to the BEAS2B cells.

### LL37 enhances IL6 release from rhinovirus-infected BEAS2B cells

**Figure 5:**
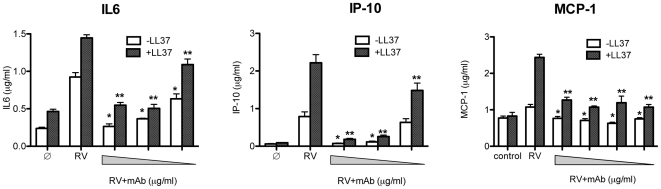
LL37 can enhance the cytokine response to Rhinovirus (RV) infection in cultured BEAS2B cells. LL37 was present at a final concentration of 3 µM and cytokine levels determined using a Milliplex cytokine/chemokine kit. The symbol ∅ denotes mock-infected cells. LL37 enhanced cytokine production in RV infected cells and the effects were blocked by increasing concentrations of a monoclonal antibody specific to TLR3 (denoted by the grey triangle; [40]). * Indicates p<0.05 compared to cytokine levels in RV infected cells while ** indicates p<0.05 compared to cytokine levels induced by addition of LL37 to RV infected cells. Each bar shows mean (±SEM; n = 3).

Rhinovirus (RV) infections activate TLR3-mediated responses in the respiratory tract, exacerbating asthma and chronic obstructive pulmonary disease [37], [38]. Moreover, TLR3 acts as an initial endosomal sensor of rhinovirus infection in human epithelial cells [39]. To determine whether LL37 can affect TLR3-mediated responses to viral infections, we infected BEAS2B cells with RV in the presence or absence of LL37. LL37 (3 µM) alone minimally induced the levels of IL6 (Figures 1B and 5), IP10 and MCP-1 by 2 ± 0.12, 1.5 ± 0.04 and 1.07 ± 0.13 fold above basal respectively (n = 3; Figure 5) while RV infection alone induced the production of IL6, IP10 and MCP-1 by 3.9 ± 0.25, 12.8 ± 0.02 and 1.31 ± 0.1 fold respectively (Figure 5; n = 3; p = 0.0001 for IL6; p<0.005 for IP10; P = 0.056 for MCP-1). The addition of LL37 (3 µM) to the infection medium enhanced IL6 production above that of RV infection by 1.6 ± 0.04 fold (p<0.002; n = 3), IP10 production by 2.8 ± 0.3 fold (p<0.005, n = 3) and MCP-1 by 2.4 ± 0.1 fold (p = 0.003, N = 3) (Figure 5). To determine whether TLR3 was responsible for the elevated cytokine production, a monoclonal antibody to TLR3, previously demonstrated to inhibit TLR3 signaling [40], decreased the enhancement of RV-induced IL6 production by LL37 by 64% ± 3.4 (Figure 5; n = 3), IP10 production by 93 ± 1% (Figure 5, n = 3) and MCP-1 by 48 ± 3% when compared to RV infection alone. These results show that LL37 can facilitate the recognition of viral infection by host cells through the activation of TLR3.

### LL37 enhances dsRNA-induced TLR3 signaling in human peripheral blood mononuclear cells (PBMCs)

**Figure 6:**
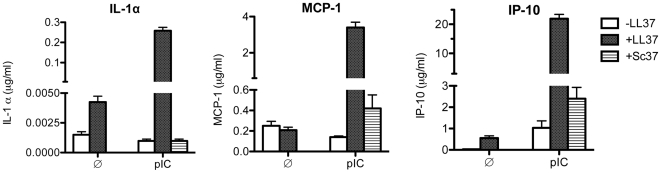
LL37 enhanced poly(I:C)-induced cytokine production in human peripheral blood mononuclear cells (PBMCs). After 24 h of poly(I:C) ±LL37 incubation, IL-1α, MCP-1 and IP-10 levels in the medium were determined using a Milliplex cytokine/chemokine kit and the amount of each cytokine normalized to the total volume of the each sample. LL37 (5.6 µM) by itself did not induce any of the cytokines measured, but significantly enhanced poly(I:C) induced production of IL-1α, MCP-1 and IP-10, while Sc37 had no effect by itself or in the presence of poly(I:C). The addition of either LL37 or Sc37 at this concentration did not affect the morphology of the cells, indicating that there was no obvious cytotoxicity (data not shown). Each bar shows the mean with one SEM (n = 3).

To determine whether LL37 enhances the response of primary cells to dsRNA, we examined cytokine release by human PBMCs (Figure 6). The culture medium of untreated human PBMCs had low levels of IL1α, MCP-1, and IP-10 (∼0.005, 0.2, and 0 µg/ml, respectively). Treatment with either LL37 (5.6 µM) or poly(I:C) (5 µg/ml) had modest effects on IL1α, MCP1 and IP-10 levels, but the addition of LL37 and poly(I:C) increased the levels of IL1α, MCP1 and IP10 by at least ten-fold above the levels seen with either poly(I:C) or LL37 alone (Figure 6; p = 0.0001; n = 3). Sc37 did not significantly increase any cytokine/chemokine levels (Figure 6; n = 3). These results demonstrate that the combination of LL37 and poly(I:C) enhances cytokine production in primary cells as it does in immortalized cell lines.

### Features of LL37 required to enhance TLR3 signaling

**Figure 7:**
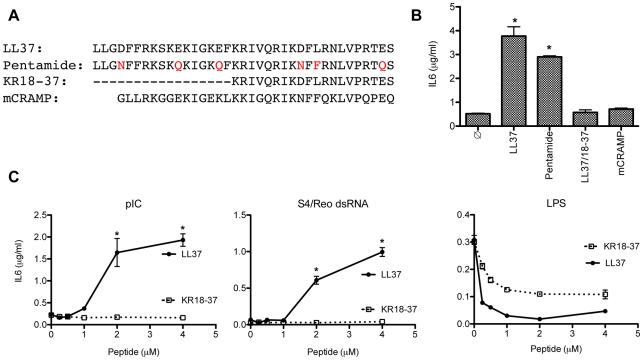
Features in LL37 required to enhance dsRNA recognition by TLR3. A) The peptides used in this set of results. Amino acid substitutions to LL37 acidic residues are shown in red. The dashes indicate that the residues are missing in the peptide KR18-37. B) The effects of the various peptides on the responses to poly(I:C) (0.13 µg/ml) in BEAS2B cells. The sample identified with a ∅ was treated with poly(I:C), but not to a peptide. Other samples were all treated with poly(I:C) and indicated peptide (2 µM for Pentamide, 4 µM for KR18-37 and 5 µM for mCRAMP). IL6 was measured 24 h after the addition of the ligands. C) The effects of LL37 or RK18-37 on IL6 levels induced by Poly(I:C), Reovirus S4 dsRNA, or LPS. *Indicates p<0.05 compared to treatment with dsRNA alone. LL37 and KR18-37 inhibited LPS induced IL6 production at all concentrations tested (p<0.05).

To define the features of LL37 required to enhance dsRNA-dependent signaling, we compared the effects of LL37 to three related peptides: Pentamide, a peptide with substitutions in multiple acidic residues in LL37, KR18-37 that lacks the first 17 residues of LL37 and is found in sweat [41], and the mouse analog of LL37 named mCRAMP (Figure 7A). Pentamide (2 µM) retained 77 ± 2% (n = 3) of the ability of LL37 to enhance poly(I:C)-induced TLR3 signaling in BEAS2B cells, indicating that the negatively-charged residues in LL37 are not critical to enhancing dsRNA-dependent IL6 production (Figure 7B). However, KR18-37 (4 µM; Figures 7B&C; p>0.5, n = 3) and mCRAMP (5 µM; Figure 6B; p>0.5, n = 3) were unable to enhance IL6 production in response to poly(I:C). Similar to LL37 (Figure S2), no cytotoxicity was detected with KR18-37 and mCRAMP at these concentrations (data not shown). Similar results were obtained when the S4 dsRNA was used as the agonist (Figure 7C). KR18-37 did retain, however, the ability to inhibit LPS-dependent signaling through the TLR4 receptor, in agreement with the results of Durr et al. [20] (Figure 7C). These results indicate that regions within LL37 that affect TLR3 and TLR4 signaling do not completely overlap.

### LL37 changes the conformation of poly(I:C) *in vitro*

**Figure 8:**
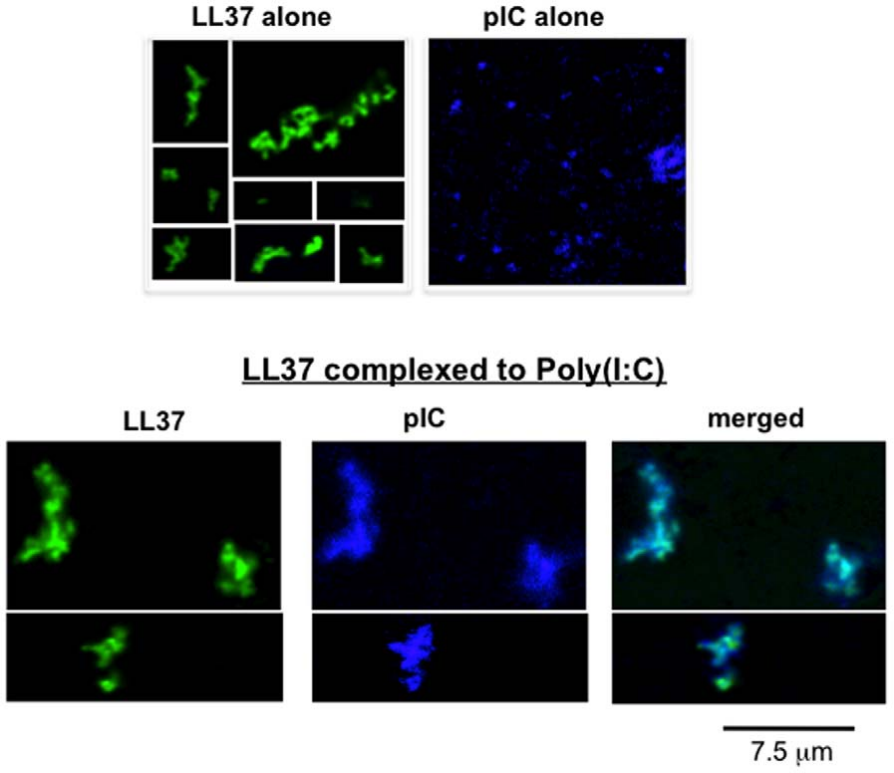
Conformations of FAM-LL37, Cy5-poly(I:C) and the complex of FAM-LL37 and Cy5-poly(I:C). Cy5-poly(I:C) (50 µg/ml) or FAM-LL37 (2 µM) was added either singly or as a mixture to coverslips. The images were obtained on a Leica TCS SP5 confocal microscope (100x objective lens). The images separated by a white line were taken from separate areas within the sample.

LL37 has been shown to bind RNAs from necrotic cells and transport self-RNAs into endosomes of dendritic cells [26]. However, it is not known whether LL37 can bind dsRNAs. To determine this, we incubated Cy5-labeled poly(I:C) (50 µg/ml) with FAM-labeled LL37 (2 µM) and visualized them on coverslips using fluorescent microscopy (Figure 8). At pH 7.4, FAM-LL37 exhibited a range of shapes including occasional filamentous structures consistent with LL37's reported ability to oligomerize [20] (Figure 8). Poly(I:C), however, appeared as primarily punctate structures with some large aggregates. When FAM-LL37 was incubated with Cy5-poly(I:C), their fluorescence extensively co-localized, indicating that LL37 can bind dsRNA (Figure 8).

**Figure 9:**
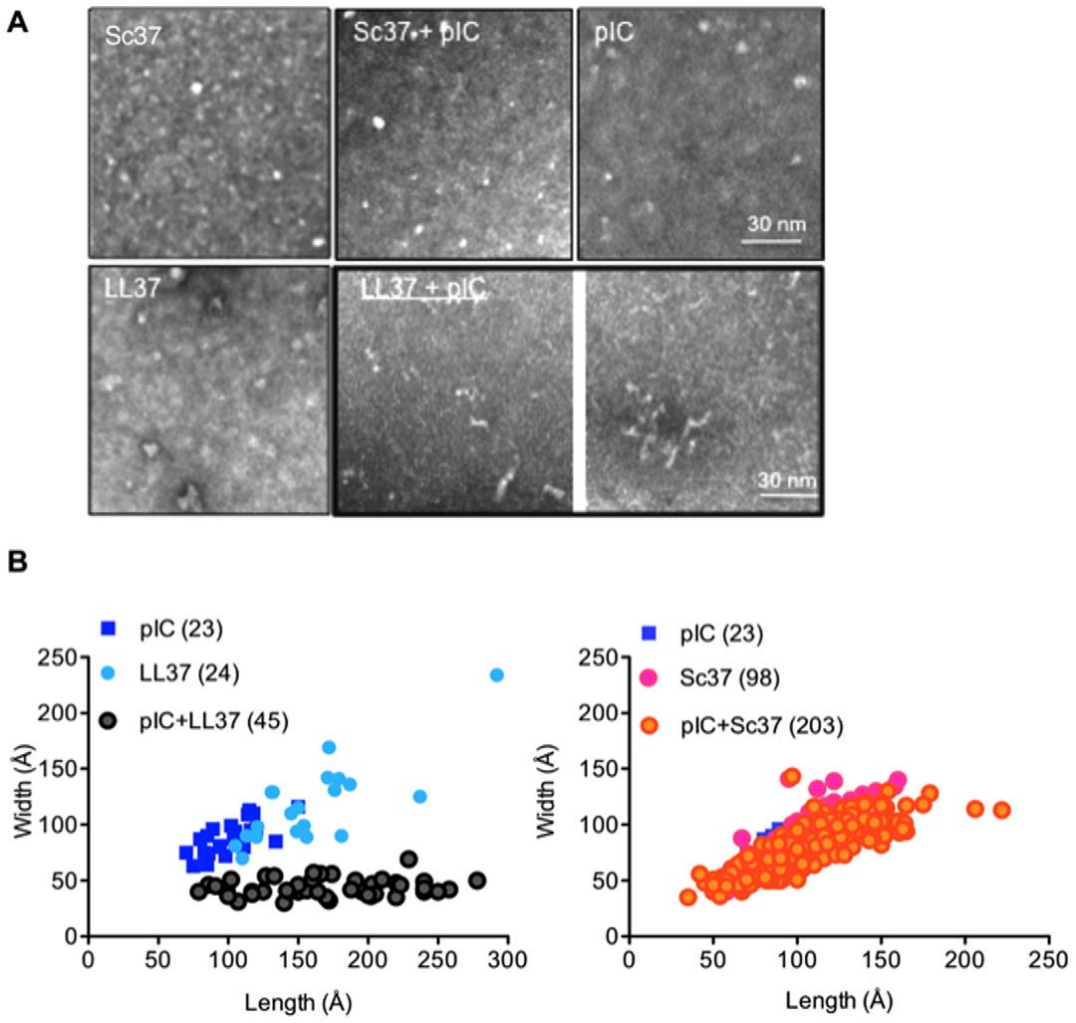
Electron microscopy of unlabeled LL37, Sc37, or poly(I:C) (pIC; 50 µg/ml) either alone or in combination. A) LL37, Sc37 or poly(I:C) was added to a carbon coated copper grid, stained with uranyl acetate and visualized using electron microscopy. The images were taken at a 40,000X magnification using a JEOL transmission microscope. LL37 and Sc37 were present at 10 µM and poly(I:C) was at 50 µg/ml. The bottom middle and right panels show images of LL37 and poly(I:C). B) A plot of the maximal lengths and widths of the subsets of particles present in the electron micrographs. Measurements were made using the toolbox within the EMAN package of software's [66]. Slopes calculated using linear regression with GrapPad Prism software. Slopes for pIC, LL37, Sc37, pIC+Sc37 are all significantly different from 0 (p<0.0005, 23–203 structures). Slope for poly(I:C)+LL37 is not significantly different from 0; p = 0.2; 45 structures). Number of particles measured for each treatment is indicated in parentheses.

To better visualize the conformation of the LL37-dsRNA complexes, we used negative-stain transmission electron microscopy to image LL37 (10 µM), size-fractionated poly(I:C) (200–500 bp; 50 µg/ml), or a mixture of the two. Sc37 (10 µM) served as a control in this experiment. The results are shown in Figure 9A. At pH 7.4, we observed heterogeneous globular structures for LL37, Sc37, and poly(I:C). Sc37 mixed with poly(I:C) showed globular structures similar to those observed with Sc37 or poly(I:C) alone. However, the mixture of LL37 and poly(I:C) resulted in a predominantly filamentous structures. The results suggest that LL37 in complex with poly(I:C) can physically alter the conformation of poly(I:C), a feature that may influence recognition by TLR3.

To quantify these structural changes, the lengths and widths of individual particles before and after the indicated treatments were determined (Figure 9B). LL37 existed primarily as small ellipsoid structures with average lengths and widths of 32 and 10 nm (slope of best fit line, 0.54 ± 0.06; R^2^ = 0.76; 24 structures). Sc37 showed similar distribution (slope of 0.8 ± 0.05, R^2^ = 0.7; 98 structures). The majority of poly(I:C) particles existed as heterogeneous particles of ca. 7–15 nm (slope, 0.53 ± 0.12; R^2^ = 0.5; 23 structures), with a small proportion that had diameters in excess of 40 nm. Poly(I:C) complexed with LL37 had an average width of 3.5 to 6 nm and lengths that ranged from 8 to 28 nm (Figure 9B; slope not significantly different from 0, p = 0.2; 45 particles). Sc37 did not cause a significant change in the structures of poly(I:C) (Figure 9B; slope of Sc37+poly(I:C), 0.5 ± 0.02; R^2^ = 0.7; 203 structures). Taken together, these results raise the intriguing possibility that LL37 could enhance poly(I:C) induction of TLR3 either by changing the conformation of the dsRNA or decreasing the oligomerization state of the dsRNAs to increase the effective concentration of poly(I:C).

We also examined poly(I:C) and LL37 with atomic force microscopy, which does not require staining the samples with heavy metals (Figure S4). The samples were placed on a graphite surface in a PBS buffer (pH 7.4). Individual poly(I:C) and LL37 adhered to the surface with comparable distributions. Furthermore, the shapes and size distributions of the individual particles are consistent with those seen in negative-stained micrographs. However, when a mixture of poly(I:C) and LL37 was placed on a graphite surface, the number of particles adhered to the surface was reduced by at least 10 fold. While this prevented us from accurately measuring particle parameters, these results suggest a significant change in the physical properties of the poly(I:C)-LL37 complex in comparison to the individual molecules.

### LL37 co-localizes with TLR3 in BEAS2B cells

**Figure 10:**
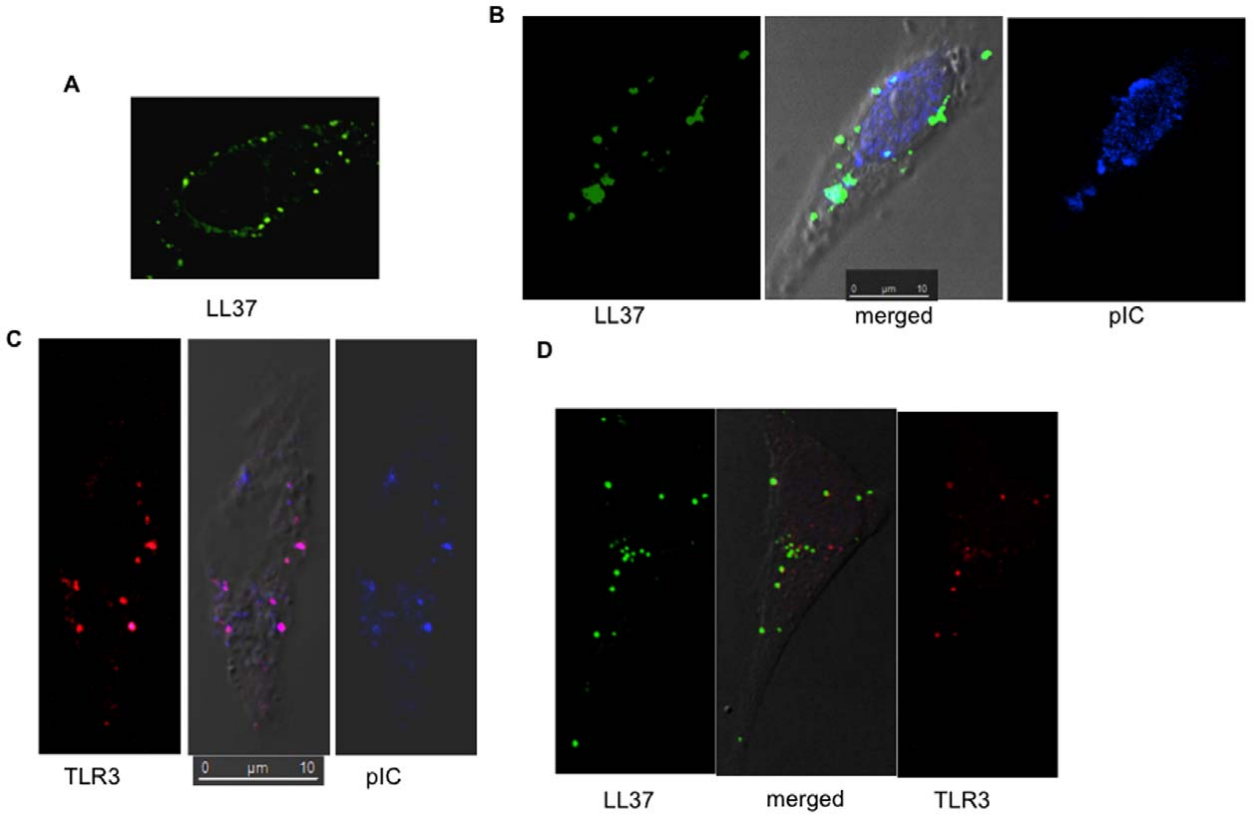
LL37 co-localizes with TLR3 and poly(I:C). A) The location of LL37 in BEAS2B cells. BEAS2B cells were cultured on coverslips and FAM-LL37 was added at a final concentration of 2 µM. Five hours after the addition of FAM-LL37, the cells were fixed and examined using confocal microscopy. B) LL37 can co-localize with Cy5-poly(I:C). FAM-LL37 (2 µM) and Cy5-poly(I:C) (1 µg/ml) were added to BEAS2B cells. The cells were fixed and imaged 5 h after the addition of the fluorescently-tagged molecules. C) TLR3 can co-localize with poly(I:C) independent of exogenously-provided LL37. BEAS2B cells were treated with Cy5-poly(I:C), then permeabilized and stained with a goat anti-TLR3 antibody in complex with Texas Red-labeled secondary antibody. D) Co-localization of FAM-LL37, endogenous TLR3 and Cy5-poly(I:C). The scale bar is 10 microns.

We next used confocal microscopy to determine whether LL37, poly(I:C) and TLR3 co-localized in BEAS2B cells. FAM-LL37 and Cy5 poly(I:C) retained bioactivity at levels comparable to unmodified counterparts (data not shown). Using confocal microscopy, FAM-LL37 (2 µM) was found inside cells 3 to 5 h after its addition to the cell culture media in the absence of poly(I:C) (Figure 10A) [42]. Cy5-poly(I:C) (0.15 µg/ml) also entered cells in the absence of exogenous LL37 (data not shown), consistent with our previous observations [43]. When both FAM-37 and Cy5-poly(I:C) were added to BEAS2B cells there was a significant overlap in the distribution of the two molecules (Figure 10B). These results indicate that both LL37 and poly(I:C) are internalized independently into BEAS2B cells and may also be internalized as a complex.

Dual label fluorescent confocal microscopy was used to determine whether TLR3 is present in the punctate structures that contain LL37 and poly(I:C). Cy5-poly(I:C) was found to colocalize with endogenous TLR3 either in the absence or the presence of LL37 (Figures 10C and 10D). These results demonstrate that LL37 and poly(I:C) can localize to endosomal compartments where TLR3 is thought to signal [43].

### Cell penetrating peptides can enhance TLR3 signaling

Next, we sought to determine if it was possible to enhance TLR3 signaling without inhibiting TLR4 signaling. Several cell-penetrating peptides (CPPs) have been documented to bind RNA, so we reasoned that they might mimic at least some of the activities of LL37 [44]. The CPP tested include the Tat peptide that is rich in basic residues, the Penetrin peptide derived from the protein antennapedia, and the T3 and T4 peptides derived from the Brome mosaic virus (BMV) capsid [45]. We examined whether these and other peptides share LL37's ability to enhance dsRNA-induced TLR3 signaling in BEAS2B or 293T/TLR3 cells. The results are presented in Table 1 as fold-enhancement by the peptides with either poly(I:C) (0.13 µg/ml) in BEAS2B cells or Reovirus dsRNA (2 µg/ml) in HEK293/TLR3 cells over signaling in the presence of dsRNA alone.

**Table 1:** Effects of peptides on TLR3 signaling in BEAS2B and HEK293T/TLR3.

Peptide	Sequence (N to C)	BEAS2B^1^	293T/TLR3^2^
LL37	LLGDFFRKSKEKIGKEFKRIVQRIKDFLRNLVPRTES	4.5 ± 0.7 (23)*	1.7±0.09 (16)*
Sc37	LLGNFFRKSKQKIGKQFKRIVQRIKNFFRNLVPRTQS	1.05±0.11 (6)	1.0±0.03 (9)
Tat47-57	YGRKKRRQRRR	3.7±0.43 (10)*	1.3±0.19 (3)
Penetrin	RQIKIWFQNRRMKWKK	2.9±1.2 (3)*	1.1±0.05 (4)
Arg(9)	RRRRRRRRR	4±0.86 (4)*	1.5±0.17 (4)*
T4	TRAQRRAAARGVQIVYKC	5.6±1.4 (6)*	1
T3	TRAQRRAAARRNRACCPGCCS	3±0.4 (8)*	1.3±0.08 (4)*
T3Ser	TSAQSSAAASSNSACCPGCCS	0.8±0.12 (4)	1
Peptide 3	TRAQRRAAARGGGVVIAC	3±0.62 (8)*	1
P22N(14-30)	NAKTRRHERRRKLAIER	1.0±0 (3)	1.3±0.14 (4)
Buforin II	TRSSRAGLQFPVGRVHRLLRK	1.0±0 (3)	1.2

1 BEAS2B cells were stimulated with 0.13 µg/ ml of poly(I:C) ± peptides (5 µM) and the amount of IL6 quantified. Fold induction above poly(I:C) induced IL6 production was calculated for each peptide. The number of experiments for each peptide is shown in parentheses, duplicate or triplicate per experiment. *Indicates p<0.05.

2 293T/TLR3 cells were stimulated with reovirus genomic RNA (2 µg/ml) and peptides (2 µM). Fold induction above Reovirus dsRNA was calculated for each peptide.

* Indicates p<0.05.

**Figure 11:**
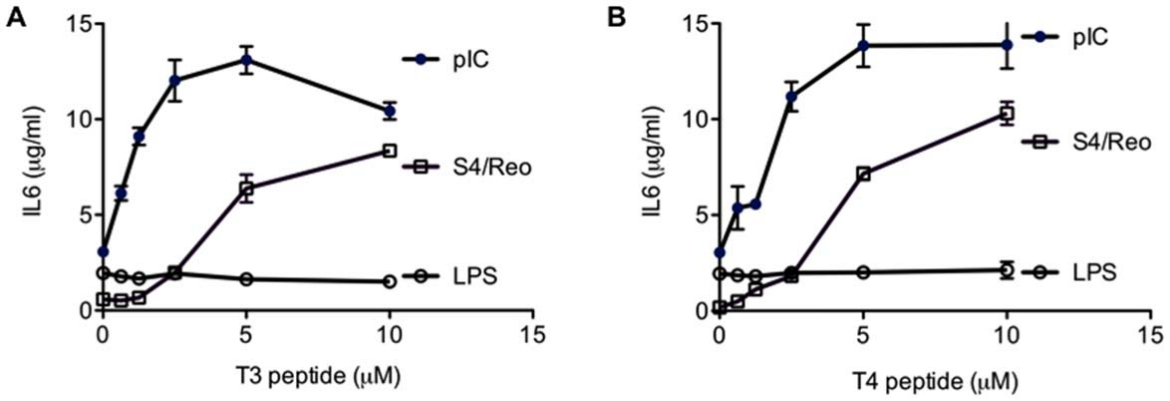
Dose-dependent enhancement of IL6 production by BEAS2B cells in response to poly(I:C) and S4dsRNA in the presence of two cell penetrating peptides. A) The T3 peptide enhanced IL6 production in the presence of dsRNAs, but not LPS. The final concentrations of the T3 peptides in each reaction are shown on the horizontal axis. Poly(I:C) and S4/Reo dsRNA were at 0.13 µg/ml and LPS was at 1 µg/ml. B) The T4 peptide also induced IL6 production in the presence of dsRNAs, but not when LPS was used as an agonist. *Indicates p<0.05 compared to treatment with dsRNA alone. Neither T3 nor T4 peptide had any effect on LPS induced IL6 level (p>0.05).

In BEAS2B cells, the Tat peptide, Penetrin, and Arg(9) all enhanced poly(I:C)-induced IL-6 production by at least 2.9-fold over the levels induced by poly(I:C) alone (Table 1; p<0.05). Peptides 3, T4 and T3 also enhanced poly(I:C)-induced signaling (Table 1 and Figure 11; p<0.05). None of the CPPs had an effect on IL6 production when added in the absence of dsRNA (data not shown). Furthermore, a variant of the peptide T3 named T3ser that had the arginines substituted with serines lost the ability to enhance IL6 production (Table 1). Interestingly, P22(14-30) [44] and Buforin II [46] which also contain a number of arginines did not enhance TLR3 signaling in BEAS2B cells (Table 1; p>0.5).

The situation was more complex in the 293T/TLR3 cells treated with Reovirus genomic RNAs. Only LL37 and Arg(9) peptide significantly enhanced reporter activity by more than 1.5-fold (p<0.05; Table 1). These results indicate that in addition to LL37, peptides that bind RNA could enhance innate immune signaling, but that the responses vary depending on the cell type, agonists and the peptides.

### Cell penetrating peptides specifically affected TLR3 signaling

To confirm that the peptides enhanced cytokine production through TLR3, cells treated with Tat peptide were subjected to siRNAs to TLR3 or siRNA to RIG-I. Knockdown of TLR3 reduced the enhancement by Tat peptide by 52 ±0.7% while siRNA to RIG-I had only a modest effect (13 ± 5.3%; data not shown). Furthermore, cells treated with EGCG, which inhibits RIG-I signaling but not TLR3 signaling [47], did not affect the Tat peptide's enhancement of IL6 production (data not shown). These results suggest that the enhancement by Tat peptide is through TLR3. Finally, we determined that IL6 levels induced by LPS were not affected by peptides T3 or T4 (Figure 11; P>0.05 at peptide concentrations from 0.6–10 µM). Similar results were observed with the Tat and Arg(9) peptides (Figure S5). Thus, some of the cell penetrating peptides that enhanced TLR3 signaling do not affect signaling by TLR4.

## Discussion

Antimicrobial peptides can regulate a number of innate immune responses [21]. In this work, we demonstrate that the antimicrobial peptide LL37 enhances signaling by TLR3 in two cell lines as well as in human PBMCs. Importantly, viral dsRNA ligands that are poor TLR3 agonists can become as potent an agonist as poly(I:C) is in the presence of LL37. LL37 also increases cytokine production in Rhinovirus-infected BEAS2B cells. In terms of mechanism, the effect of LL37 requires dsRNA and is likely to increase TLR3 signaling rather than to activate TLR3 gene expression. LL37 also modifies the conformation of poly(I:C), a feature that could impact ligand recognition by TLR3. Finally, we demonstrated that several peptides previously classified as cell-penetrating peptides and are known to bind RNA enhance TLR3 signaling without affecting LPS (TLR4)-dependent signaling.

The role of LL37 and dsRNA-binding peptides in TLR3 signaling could resolve disparate observations in the TLR3 field. We have consistently observed that viral dsRNAs are poor TLR3 agonists by themselves (Figure 3C). While mRNAs from necrotic cells and even siRNAs have been reported to be agonists for TLR3 [15], [16], [26], these RNAs have no effect on TLR3 signaling in BEAS2B cells or HEK293T cells overexpressing TLR3 (Kao, unpublished observations). Since TLR3 is activated during viral infection (Figure 5), additional co-factors may be needed to enhance the ability of TLR3 to recognize viral dsRNAs during infection. In this study, we found that LL37 enhances the recognition of viral dsRNA by TLR3. It is possible that LL37, or similar endogenous co-factors, are missing in highly purified RNAs and hence these RNAs could not induce TLR3 signaling. Moreover, the responses may be dependent on the cell type. Even in the two cell lines we used, LL37 had different effects. In BEAS2B cells, LL37 enhances TLR3 signaling induced by either poly(I:C) or viral dsRNA. However, in 293T/TLR3 cells, LL37 only enhanced TLR3 signaling induced by viral dsRNAs and not by poly(I:C). Furthermore, some cell penetrating peptides can mimic the activities of LL37 and we observed that they had differential effects between the two cell lines.

The current study describes a pharmacological role for LL37 in enhancing dsRNA dependent TLR3 signaling. However, it is likely that endogenously released LL37 may have a physiological role in activating TLR3 during viral infection for the following reasons: LL37 is generated from hCAP-18 by proteolysis. Basal levels of LL37 are undetectable to low in many cell types, including airway epithelial cells [48] and BEAS2B cells (Lai, unpublished observations). It is induced during bacterial [49] and viral infection [50] or by Vitamin D analogs [49], [51]. Concentrations of LL37 range from 3 µM in bronchioalveolar lavage fluid from patients with cystic fibrosis [52] to 40 µM in neutrophil granules [53] to 304 µM in psoriatic lesions [54]-at or higher than the LL37 concentrations used in the current study. Leukotriene B4 increases LL37 secretion from neutrophils and decreases viral load in mice after influenza infection [55]. Antibodies to LL37 attenuate this effect, suggesting that endogenously released LL37 plays an important role in defense against viral infections [55]. LL37 also reduces viral loads after vaccinia virus infection while CRAMP (the murine LL37 analog) knockout mice showed increased vaccinia pox formation [56]. In this study, we found that Rhinovirus infection increases the release of cytokines (also see [57]) and that addition of LL37 enhances this TLR3-dependent response. Further studies will be needed to determine the physiological source(s) of LL37, whether rhinovirus infection increases hCAP18 transcription and secretion of LL37 from BEAS2B cells, and whether LL37 is released from other cells such as neutrophils upon viral infection. It is possible that, while addition of LL37 protein enhances TLR3 signaling in our studies, other, as yet unidentified endogenous factor(s) enhance viral sensing by TLR3 during viral infection. To address the source and identity of these factors and to determine whether LL37 is involved, BEAS2B cells could be co-cultured with neutrophils before or after viral infection and the level of LL37 quantified in the co-culture medium with or without prior treatment of siRNAs to LL37. Finally, rhinovirus infection activates TLR3 in BEAS2B cells in the absence of exogenous dsRNA ligands, suggesting that TLR3 recognizes intracellular viral or cellular RNAs, as would be the case during viral infection.

LL37 by itself has been proposed to modulate cytokine responses by a number of mechanisms. These include activation of the P2X7 receptor, transactivation of the epidermal growth receptor and activation of MAP/ERK and p38 pathways [58]. Filewood [28] also reported that there is significant cytotoxicity in association with LL37 and poly(I:C), indicating that there could be an activation of TLR3 through the cellular stress responses. We concur that high concentration of LL37 (higher than concentrations we used in this study) can lead to decreased cell viability. However, the rapid induction of IL6 message and protein production we observed suggests that an early response to LL37 and dsRNA is activation of TLR3 signaling. LL37 co-localizes with dsRNA in endosomes, where TLR3 is presumed to signal. We have demonstrated that tyrphostin AG 1478, a specific inhibitor of the EGFR, had no effect in BEAS2B cells on IL6 production induced by either poly(I:C) or poly(I:C) in combination with LL37 (Lai Y, unpublished observations), suggesting that the enhancement of IL6 secretion by LL37 is not mediated by EGFR transaction. Moreover, since TLR3 also signals through the p38/MAP kinase cascade [31], inhibitors of this pathway would also be expected to block the effects of LL37 on dsRNA induction of TLR3 signaling. Since poly(I:C) alone or poly(I:C) in combination with LL37 significantly induced secretion of a number of cytokines, there may be a secondary induction by these inflammatory cytokines on epithelial and neighboring cells. Therefore, to fully understand the mechanism by which LL37 activates TLR3, it will be important to distinguish primary and secondary effects.

We show in this study that LL37 interacts directly with dsRNA. While LL37 has been reported to bind complex mixtures of self-DNAs and self-RNAs to modulate TLRs7-9 [25], [26], it is not known how ligand binding modulates TLR7-9 and whether the same mechanisms are used to modulate signaling by TLR3. Long ssRNA and dsRNAs primarily exist as globular structures [59]. We have observed that LL37 affected the response of cells to dsRNA (and not to several ssRNAs, including the JFH-1 genomic RNA). This suggests that there will be significant differences in how LL37 modulates responses to single and double-stranded RNAs. Particularly intriguing to us is that LL37 can alter the conformation of poly(I:C). This result may be meaningful in light of the observation that the X-ray structure of TLR3 with poly(I:C) demonstrates that a dimer of the TLR3 ectodomain is complexed to a linear poly(I:C) [14]. We note that RIG-I has ATPase activity that is correlated with response to at least some dsRNA ligands [60], [61], [62]. TLR3 lacks helicase activity and may require factors such as LL37 to facilitate TLR3 signaling by unwinding the dsRNA, thus allowing binding and recognition of the dsRNA by TLR3. Given that poly(I:C) does not apparently require LL37 to serve as an agonist for TLR3, there must exist differences in the secondary and/or higher order structures of viral dsRNAs that require factors such as LL37.

Although LL37, dsRNA, and TLR3 are found in endosomes in BEAS2B cells, at present we do not know whether LL37 participates in the signaling complex. In dendritic cells, LL37 binds to and protects self-RNA from enzymatic degradation, facilitating the transport of self-RNA into endocytic compartments to activate TLR7/8 [26]. LL37 may function similarly in epithelial cells to facilitate the trafficking of the dsRNAs to enhance TLR3 signaling. This aspect of the mechanism for TLR3 signaling will require analysis that is beyond the scope of this study. Preliminary results suggest that mCRAMP, which does not activate TLR3 (Figure 7B), does not co-localize with TLR3 in endosomes, suggesting that the trafficking of LL37 into endosomes containing TLR3 requires specific pathways.

Finally, the identification of CPPs as enhancers of dsRNA-dependent TLR3 signaling has a number of implications. For example, these peptides allow the enhancement of TLR3 signaling without a concomitant effect on LPS-dependent responses. Moreover, CPPs complexed to viral or cellular dsRNAs may affect the response to viral infection and potentially impact autoimmunity. These results not only further our understanding of how TLR3 could be activated by physiologically relevant ligands like viral dsRNAS, but also will aid in our design of better and more selective TLR therapeutic modulators.

## Materials and Methods

### Reagents

Poly(I:C) (Amersham Biosciences or GE Healthcare) was reconstituted in phosphate buffered saline (PBS; Invitrogen, Carlsbad, CA). Size-selected Poly(I:C) of 50–125 bps were generated as described previously [35]. Fluorescently-labeled Cy5-poly(I:C) was made as described in [43]. LPS was from Sigma-Aldrich Inc. DsRNAs from Reovirus were extracted from purified virions (a kind gift from Dr. Danthi, Indiana University). The Reovirus S4 dsRNA was synthesized by *in vitro* transcription from two cDNAs, each of which had a T7 promoter to produce the sense or the antisense ssRNAs. The ssRNAs were then adjusted to a 1∶1 molar ratio in PBS and heated to 95°C for five minutes followed by slow cooling to room temperature to allow the strands to anneal to form S4 dsRNA. Plant endornavirus BPEV dsRNA was the kind gift of R. Valverde (Louisana State University, Baton Rouge, LA). Positive-strand ssRNA from hepatitis virus JFH1 was made by *in vitro* transcription from a cDNA of JFH-1 [63]. Plasmids encoding the wild-type TLR3, TLR4 and RIG-I were from InvivoGen (San Diego, CA). LL37, scrambled LL37 (Sc37), 5′ fluorescein-labeled LL37 (FAM-LL37) and cell penetrating peptides were from Anaspec (Fremont, CA) or custom synthesized by Peptide 2.0 (Chantilly, VA) and stored at −20°C until use. The anti-human TLR3 monoclonal antibody was previously described by Teng et al. [40].

### Cell culture

The SV40 transformed human bronchial epithelial cell line, BEAS2B (ATCC), was cultured in BEGM media with supplements (Lonza, Basel, Switzerland). HEK293T cells (ATCC) were cultured in DMEM supplemented with 10% FBS (Invitrogen). Human PBMCs were isolated from whole blood collected from healthy donors and processed as described [43]. All necessary permissions, licenses, approvals and handling of biological samples were according to protocols developed by Centocor, R&D, Inc.

### Quantification of cytokine/chemokine release by BEAS2B and PBMCs

BEAS2B cells were plated at 1.5×10^4^ cells/well in 96-well plates. 24 h after plating, the media was replaced with 50 µl of media containing the indicated treatments. Unless otherwise noted, poly(I:C) and LPS were added to the medium of cells to final concentrations of 0.13 µg/ml and 1 µg/ml, respectively. To activate RIG-I, 10 nM shR9 with a 5′ triphosphate was transfected into cells using Lipofectamine 2000 (Invitrogen, Carlsbad, CA). Supernatants were collected after 18-24 h and assayed for IL6 or IL8 secretion using an ELISA kit according to the manufacturer's protocol (BD Biosciences, San Diego, CA). The IL6 or IL8 level was calculated based on a standard curve fitted using a sigmoidal variable slope dose-response using software from GraphPad Prism (La Jolla, CA) and normalized to µg/ml per well. For PBMCs, an initial 150,000 cells per well were suspended in 100 µl of RPMI medium (Invitrogen) containing 10% FBS and incubated overnight at 37°C, 5% CO_2_. The cells were treated with poly(I:C) at 5 µg/ml in the absence or presence of LL37 (5.6 µM) for 20–24 h. This level of LL37 did not obviously affect PBMC morphology. The supernatants were collected and frozen until analysis. Cytokine levels for IP10, IL1α and MCP1 were measured using a Milliplex assay kit (Millipore, Billerica, MA).

### siRNA knockdown of TLR3 and RIG-I

BEAS2B cells were seeded at 1×10^5^ cells/well in a 48-well tissue culture plate or 1.0×10^4^ cells/well in a 96-well plate in BEGM media. 4-6 h after plating, cells were transfected with control siRNA (non-targeting sequence, Santa Cruz Biotechnology, CA), a pool of three siRNAs specific to TLR3 (Santa Cruz Biotechnology) or siRNA to RIG-I (Qiagen, Valencia, CA). Cells were treated with agonists 48 h later and the supernatant measured for IL6 production as described above.

### RT-PCR to determine mRNA levels for IL6, IFNβ and TLR3

Primer sets: TLR3 (forward, 5′-GATCTGTCTCATAATGGCTTG-3′; reverse: 5′-GACAGATTCCGAATGCTTGTG-3′ [64]; IL6 (forward, 5′-CACAGACAGCCACTCACCTC-3′, reverse, 5′ AGCTCTGGCTTGTTCCTCAC-3′ [64]); IFNβ primers (forward: 5′-TGCTCTCCTGTTGTGCTTCTCC-3′; reverse, 5′- CATCTCATAGATGGTCAATGCGG-3′ [65]) and GAPDH primers (5′-GAGTCAACGGATTTGGTCGT-3′; reverse: 5′-TGGGATTTCCATTGATGACA-3′ [64]). Total RNA (pooled from either six wells of a 96-well plate or three wells of a 48-well plate) was extracted by RNeasy (Qiagen, Valencia, CA) and digested by DNase I (Qiagen). 0.5 µg of total RNA was then reverse transcribed to cDNA with MMLV reverse transcriptase (Ambion, Austin, TX) using random decamers (New England Biolabs, Ipswich, MA). Real-time RT-PCR was performed with a dsDNA-binding dye, SYBR Green (Bio-Rad, Hercules, CA) with an initial 3 min denaturing temperature of 95°C, followed by a total of 40 cycles of 30s of denaturation at 95°C and 30s of annealing at 55°C and elongation at 72°C. The expression levels of mRNA were normalized by the median expression of a housekeeping gene (GAPDH). Each sample was analyzed in duplicates or triplicates. Treatments with agonists were calculated as fold above relative expression of mRNA above control (no treatment). TLR3 mRNA levels for each treatment were calculated as %TLR3 mRNA for samples treated with control nonspecific siRNA.

### Rhinovirus infection of BEAS2B cells

BEAS2B cells were plated at 5×10^4^ cells/well and grown overnight at 37°C, 5% CO_2_. The media were removed and replaced with 90 µl fresh medium/well. Cells were then infected with Rhinovirus (ATCC, catalog number VR-283, 0.5–1×10^5^ pfu/well) in the absence or presence of LL37 (3 µM). Supernatants were collected after 24 h incubation at 37°C and the level of IL6 determined.

### TLR3 luciferase reporter assay

Luciferase reporter assays were performed according to the protocol of [36]. Briefly, HEK 293T cells were plated in white-walled 96-well plates at 4.4×10^4^ cells/well and transfected with plasmids pUNO-huTLR3 (0.5 ng/well), pISRE-Luc (30 ng/well) and phRL-TK (5 ng/well) using Lipofectamine 2000. The cells were grown for 24 h to allow expression from the plasmids. Poly(I:C) (1 µg/ml) with or without peptides was added to sets of transfected cells to induce TLR3-dependent ISRE activity. After 18–24 h of incubation, luciferase activity was determined using the Dual-Glo Luciferase assay system (Promega).

### Electron microscopy of LL37 and poly(I:C)

Poly(I:C) at 50 µg/ml (low molecular weight, Invitrogen) was incubated alone or with LL37 or Sc37 (each at 10 µM) in Tris-buffered saline, pH 7.4 at 4°C for 15 min. Sample of 10 µl was placed on top of a glow-discharged 400 mesh carbon-coated copper grid (EMS). After one min incubation at RT, the drop was removed by absorption onto filter paper. The grids containing the samples were then stained with 2% uranium acetate for 20 s. Excess stain was removed and the grids were air-dried for 15 min. Micrographs of the grids were taken with JEOL transmission microscope at 80 kV, using 40,000x magnification. The micrographs were collected and measurement of length and width of particles were calculated using EMAN software as described by [66]. Data points for each treatment were fitted using linear regression and the slope of each line was calculated using GraphPad Prism software.

### Fluorescence microscopy of FAM-LL37 and Cy5-poly(I:C)

2 µM FAM-LL37 or 50 µg/ml Cy5-poly(I:C) was added individually or together in PBS (pH 7.4) with 50 mM NaCl. After 1 h, the samples were spotted on coverslips followed by fixing with 4% paraformaldehyde and three washes with PBS. The coverslips were mounted in mounting medium with DAPI (Vector laboratories, Burlingame, CA). Images were obtained with a Leica TCS SP5 scanning confocal microscope using an HCX PL APO Lambda Blue 100x oil objective (Leica Microsystems, Bannockburn, IL). Excitation was at 20% of the maximum laser power. The images were captured with a scanning speed of 200 Hz and image resolution of 512×512 pixels and analyzed using Leica Application Suite 2.02.

For subcellular localization studies, BEAS2B cells were seeded on coverslips and incubated for 24 h before use. FAM-LL37 (2 µM) was added in the absence or presence of 1 µg/ml Cy5-poly(I:C) and incubated at 37°C for another 5 h. The cells were then fixed with 4% paraformaldehyde at RT for 30 min and permeabilized with 0.1% Triton X-100 at RT for 15 min. All major steps in the localization procedure were delineated by three washes with PBS. After the addition of blocking buffer (1% BSA in PBS) for 1 h at RT, the cells were incubated overnight at 4°C with goat anti-TLR3 antibody (3 µg/ml; R&D Systems, Minneapolis, MN) prior to the addition of a Texas Red-labeled anti-goat mAb for 1 h at RT (Santa Cruz biotechnology). The coverslips were mounted in mounting medium and imaged as described above using 63x magnification.

### Statistical analysis

Depending on the experiment, statistical analysis is either *t*-test or ANOVA followed by Dunett's multiple comparison post-tests using statistical analysis software provided by GraphPad Prism. P<0.05 was considered statistically significant.

## Supporting Information

Figure S1
**LL37 (3 µM) in the presence of poly(I:C)(pIC; 0.13 µg/ml) induces the synthesis of the IL6 and IFNβ message 20 h after its addition to the medium of BEAS2B cells.** mRNA levels for IL6 and IFNβ were determined by real time RT-PCR using specific primers. * Indicates p<0.05 compared to no treatment (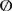) and ** indicates p<0.05 compared to treatment with poly(I:C). These results show that LL37 induces the genes predicted to be transcribed in response to TLR3 signaling.(TIF)

Figure S2 **Effects of LL37 concentration on viability of BEAS2B cells±poly(I:C).** Cell viability was assessed using the WST-1 assay (Clontech, Mountain View CA). After treatments, cells were incubated with WST-1 substrate for 4 h and absorbance was measured at 450 nM with 630 nm as reference. The readout performed in the absence of LL37 (i.e. Control) is defined as 100%. At up to 5 µM, LL37 has no effect on cell viability in the absence of poly(I:C). In the presence of poly(I:C), 3 µM LL37 did not show significant toxicity (P>0.5). The graph is representative of five experiments. These results show that the concentration of LL37 (3 µM) used in our experiments with BEAS2B cells do not have obvious cytotoxicity.
(TIF)

Figure S3 **SiRNA to RIG-1 decreases RIG-I message in BEAS2B cell.** A) A comparison of the amount of the RIG-I message in cells treated with siRNAs to RIG-I normalized to the results from cells treated with a nonspecific siRNA. Message abundance was determined by RT-PCR. B) Level of IL6 produced by BEAS2B cells exposed to three different ligands. The cells were transfected with the siRNAs 48 h prior to the transfection of the RIG-I agonist shR9 (10 nM) or addition of poly(I:C) (0.13 µg/ml) or LPS (1 µg/ml) to the cell media. These results support our claim that LL37-induced changes in the innate immune response are mediated by TLR3.
(TIF)

Figure S4 **Atomic force microscopy image of poly(I:C), LL37, and a 1∶10 complex of the two.** Each imaged area corresponds to a representative 1 mm×1 mm area. The samples were absorbed onto a freshly peeled graphite surface. These results show that poly(I:C) and LL37 exist in higher order structures and confirm the results from negative-stained electron micrographs. However, the mixture of poly(I:C) and LL37 did not absorb well onto the graphite surface. This indicates that the complex likely has significantly different chemical properties than that of either poly(I:C) or LL37 alone.
(TIF)

Figure S5 **The LL37 (3 µM), Tat and Arg(9) peptides (10 µM) enhance poly(I:C) (0.13 µg/ml)-dependent IL6 production without a significant effect on LPS-dependent IL6 production (at 1 mg/ml).** *Indicates p<0.05 compared to treatment with poly(I:C) alone (Ø). **Indicates p<0.05 compared to treatment with LPS alone (Ø). Tat or Arg(9) has no effect on LPS signaling (p>0.5). These results show that cell-penetrating peptides can mimic the activity of LL37 in enhancing TLR3 signaling but do not share LL37's ability to inhibit TLR4 signaling.
(TIF)
