# Supplementary figures and images for: LL37 and Cationic Peptides Enhance TLR3 Signaling by Viral Double-stranded RNAs
Source: PLoS One. 2011 Oct 21;6(10):e26632. doi: 10.1371/journal.pone.0026632 (PMC3198786; doi:10.1371/journal.pone.0026632)

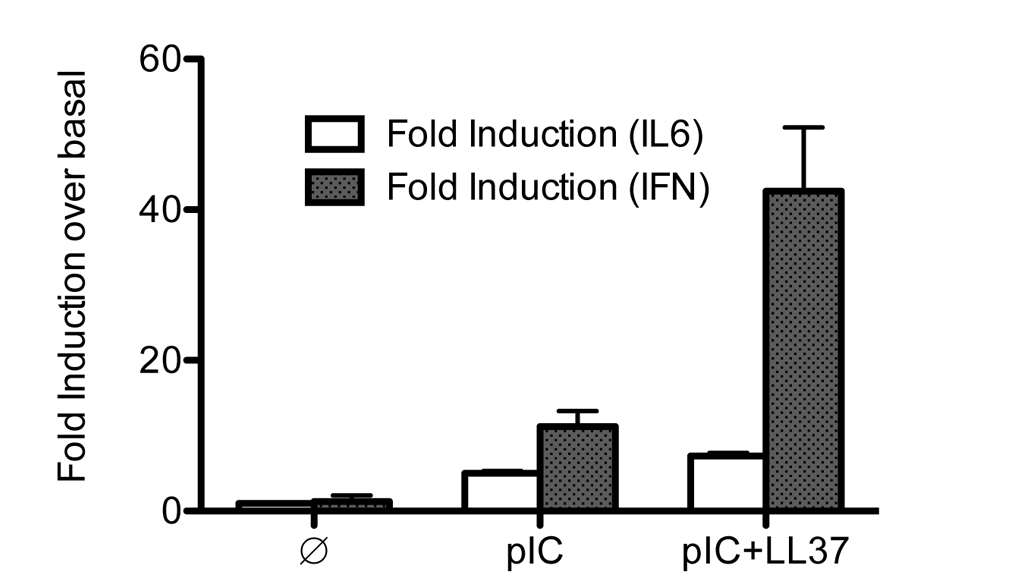

Supplement: Figure S1 — LL37 (3 µM) in the presence of poly(I:C)(pIC; 0.13 µg/ml) induces the synthesis of the IL6 and IFNβ message 20 h after its addition to the medium of BEAS2B cells. mRNA levels for IL6 and IFNβ were determined by real time RT-PCR using specific primers. * Indicates p<0.05 compared to no treatment () and ** indicates p<0.05 compared to treatment with poly(I:C). These results show that LL37 induces the genes predicted to be transcribed in response to TLR3 signaling. (TIF) [file pone.0026632.s001.tif]

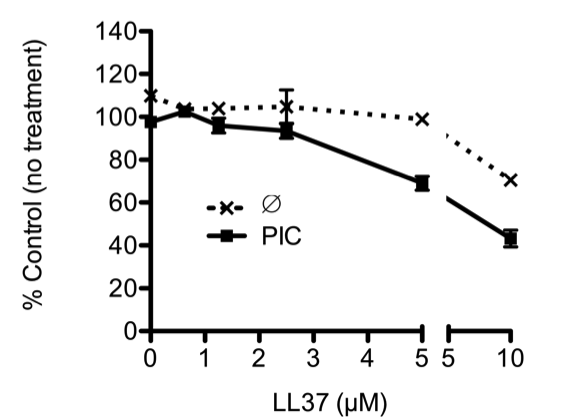

Supplement: Figure S2 — Effects of LL37 concentration on viability of BEAS2B cells±poly(I:C). Cell viability was assessed using the WST-1 assay (Clontech, Mountain View CA). After treatments, cells were incubated with WST-1 substrate for 4 h and absorbance was measured at 450 nM with 630 nm as reference. The readout performed in the absence of LL37 (i.e. Control) is defined as 100%. At up to 5 µM, LL37 has no effect on cell viability in the absence of poly(I:C). In the presence of poly(I:C), 3 µM LL37 did not show significant toxicity (P>0.5). The graph is representative of five experiments. These results show that the concentration of LL37 (3 µM) used in our experiments with BEAS2B cells do not have obvious cytotoxicity. (TIF) [file pone.0026632.s002.tif]

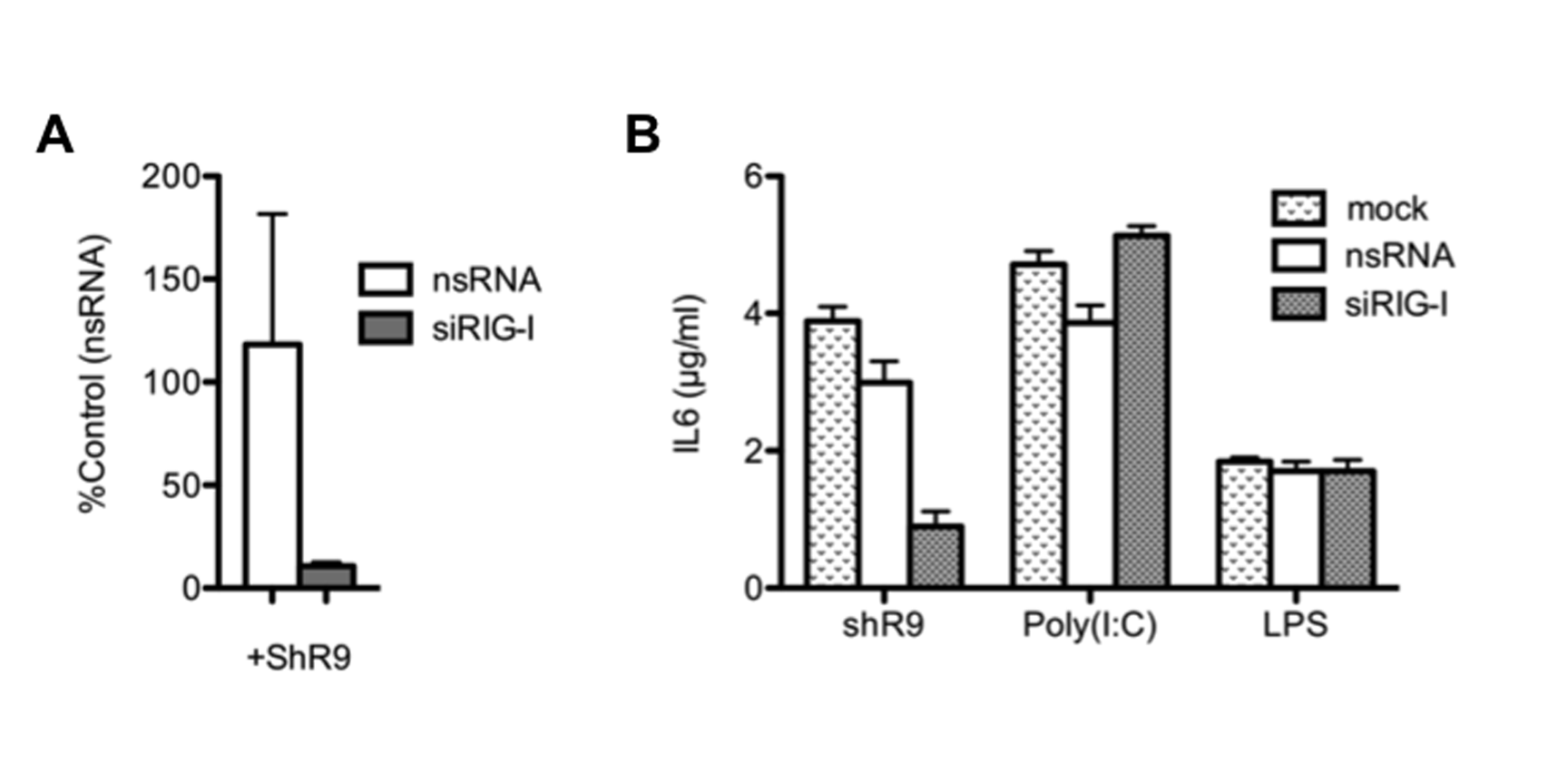

Supplement: Figure S3 — SiRNA to RIG-1 decreases RIG-I message in BEAS2B cell. A) A comparison of the amount of the RIG-I message in cells treated with siRNAs to RIG-I normalized to the results from cells treated with a nonspecific siRNA. Message abundance was determined by RT-PCR. B) Level of IL6 produced by BEAS2B cells exposed to three different ligands. The cells were transfected with the siRNAs 48 h prior to the transfection of the RIG-I agonist shR9 (10 nM) or addition of poly(I:C) (0.13 µg/ml) or LPS (1 µg/ml) to the cell media. These results support our claim that LL37-induced changes in the innate immune response are mediated by TLR3. (TIF) [file pone.0026632.s003.tif]

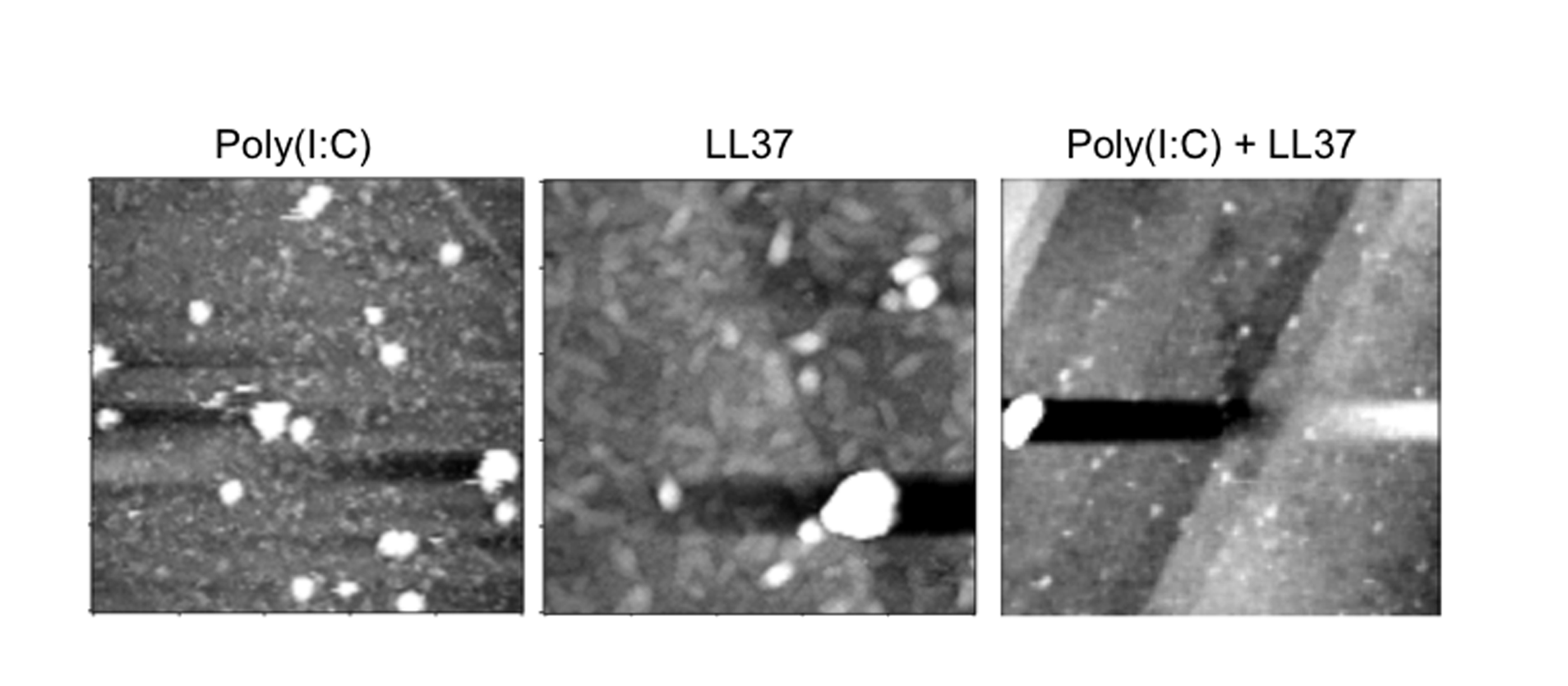

Supplement: Figure S4 — Atomic force microscopy image of poly(I:C), LL37, and a 1∶10 complex of the two. Each imaged area corresponds to a representative 1 mm×1 mm area. The samples were absorbed onto a freshly peeled graphite surface. These results show that poly(I:C) and LL37 exist in higher order structures and confirm the results from negative-stained electron micrographs. However, the mixture of poly(I:C) and LL37 did not absorb well onto the graphite surface. This indicates that the complex likely has significantly different chemical properties than that of either poly(I:C) or LL37 alone. (TIF) [file pone.0026632.s004.tif]

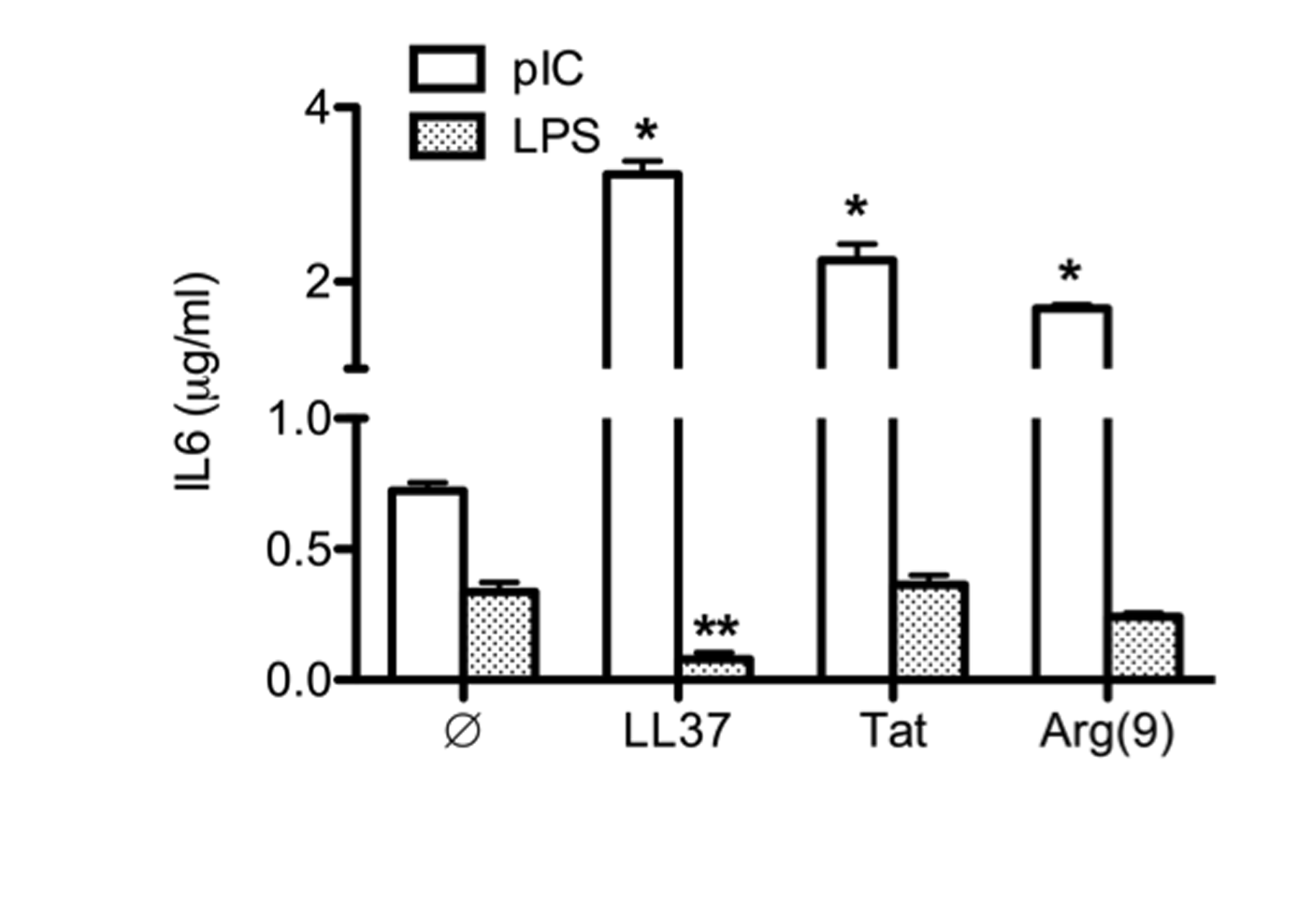

Supplement: Figure S5 — The LL37 (3 µM), Tat and Arg(9) peptides (10 µM) enhance poly(I:C) (0.13 µg/ml)-dependent IL6 production without a significant effect on LPS-dependent IL6 production (at 1 mg/ml). *Indicates p<0.05 compared to treatment with poly(I:C) alone (Ø). **Indicates p<0.05 compared to treatment with LPS alone (Ø). Tat or Arg(9) has no effect on LPS signaling (p>0.5). These results show that cell-penetrating peptides can mimic the activity of LL37 in enhancing TLR3 signaling but do not share LL37's ability to inhibit TLR4 signaling. (TIF) [file pone.0026632.s005.tif]
